# Healthwashing in high-sugar food advertising: the effect of prior information on healthwashing perceptions in Austria

**DOI:** 10.1093/heapro/daaa086

**Published:** 2020-12-08

**Authors:** Raffael Heiss, Brigitte Naderer, Jörg Matthes

**Affiliations:** 1 Center for Social and Health Innovation, Management Center Innsbruck (MCI), Universitätsstraße 15, 6020 Innsbruck, Austria; 2 Department of Media and Communication, Ludwig-Maximilians-University Munich, Oettingenstrasse 67, 80538 Munich, Germany; 3 Department of Communication, University of Vienna, Waehringer Straße 29, 1090 Vienna, Austria

**Keywords:** healthwashing, health information, nutritional information, health literacy, food advertisement

## Abstract

In the context of exceeding levels of sugar consumption, some food companies advertise high-sugar products using inappropriate and misleading health claims (i.e. healthwashing). To reduce sugar consumption, consumers need to recognize what these healthwashed claims are. This study investigates how prior sugar-related health information moderates the effect of exposure to healthwashed advertisements (ads) on healthwashing perceptions and how such perceptions are related to attitudes towards product consumption. We conducted a 2 × 2 online experiment with 292 adult participants in Austria. We manipulated the presence of healthwashing and participants’ prior sugar-related health information. The results indicated that exposure to healthwashed ads increased healthwashing perceptions *only* when the participants received additional health information prior to ad exposure, whereas no significant effect was found when the participants did not receive such prior health information. Healthwashing perceptions were then negatively related to individuals’ attitudes towards product consumption. Based on these results, the study suggests that public access to health-related information might play an important role in empowering consumers to detect inappropriate health claims and become more critical towards food companies’ underlying strategies in ads.

## INTRODUCTION

Existing research indicates that people in western societies consume too much added sugar and that added sugar in processed food is a key driver of obesity and type 2 diabetes (Malik *et al.*, 2010; Evans, 2017). A recent report from the World Health Organization (WHO, 2015) suggests that humans should consume <10% (and preferably even <5%) of their total energy intake from added or free sugar (the latter also includes naturally occurring sugars in honey, syrups or fruit juice). This is equivalent to <50 g (preferably 25 g) of sugar intake each day (for a person with a healthy body weight, consuming about 2000 kcal/day). However, in reality, sugar consumption is much higher than these reference values. For instance, sugar intake is reported to be 126 g sugar per day in the USA, 93 g in the UK and 88 g in Austria (Euromonitor data reported in Ferdman, 2015).

One key driver of sugar consumption is advertisements (hereafter: ads) promoting products with high levels of added sugar. In the context of increasing health awareness, some companies try to mislead consumers by framing these high-sugar products as healthy, for instance, by providing cues to sports celebrities, sports activities or health in general (Dixon *et al.*, 2011; Bragg *et al.*, 2018). This practice of using misleading and inappropriate claims about the health impact of products is called ‘healthwashing’. We define healthwashing as *the strategy of presenting genuinely unhealthy products in a misleading context of fitness, sports or other activities related to a healthy lifestyle.* One key strategic goal of healthwashing is an image transfer from sports events or celebrities to the company and the brands and products they offer (Bragg *et al.*, 2018). In other words, consumers are guided to inherently associate unhealthy products with health and energy rather than with the potential negative health effects of products high in sugar, salt and/or fat. For example, Coca Cola is an established partner of the international football league FIFA, and the German national football team heavily advertised Coca Cola in TV spots.

Existing research indicates that the share of such misleading health claims in ads is increasing (Whalen *et al.*, 2018) and that misleading health claims may influence attitudes and consumption behaviour (e.g. Klepacz *et al.*, 2016). In this context, better informed consumers might be less susceptible to such claims, for they are more capable (Grunert *et al.*, 2011; Dallacker *et al.*, 2018) and self-efficacious (Parmenter *et al.*, 2000; Verbeke, 2008) to judge the nutritional quality of advertised food products. However, we still know little about the preconditions under which individuals are able to detect healthwashing (i.e. misleading health claims) in advertising. Moreover, to our knowledge, there is no research on how prior health information may affect individuals’ ability to detect healthwashing and how healthwashing perceptions may affect future food consumption. This study contributes to fill these research gaps using an experimental approach.

## LITERATURE REVIEW

### Healthwashing and healthwashing perceptions

Consumers are increasingly exposed to a myriad of health-related ads (Tian and Robinson, 2009; Boelsen-Robinson *et al.*, 2016). The largest share of all advertised food products is composed of unhealthy (but profitable) processed foods (Manganello *et al.*, 2013; No *et al.*, 2014; Akçil Ok *et al.*, 2015; Freeman *et al*., 2016). At the same time, food companies are confronted with increasing levels of health consciousness and stronger demands for healthy products (Kroger *et al.*, 2006; Kriwy and Mecking, 2012). By using health claims in ads for unhealthy products, marketing is speaking to these needs. As a result, consumers may become misled about the nutritional values of advertised foods. In this context, it is crucial that consumers are informed about nutrients (e.g. sugar) added to foods and how this processing may affect their health. Only if these preconditions are met will they critically appraise health-related information and thus understand and detect inappropriate health claims put forth in ads (Nutbeam, 2000; Sørensen *et al.*, 2012; Truman *et al.*, 2020). In this study, we measure the detection of healthwashed claims as *healthwashing perceptions (or perceived healthwashing)*, defined as *the belief that the explicit or implicit health claims put forth in ads do not match the advertised product’s actual health properties.*

This practice of misleading consumers has also been observed in the marketing of environmentally unfriendly products or services, like airline flights. Such claims can occur as false, incomplete or inaccurate information, leading to misinterpreting a product as eco-friendly (Laufer, 2003). In a similar vein, food companies use health claims to advertise highly processed foods. This is problematic because if consumers are frequently exposed to ads that link unhealthy products with misleading health cues, they may accept products in their daily diet that they would hardly choose based on these products’ actual nutritional qualities. This is because advertising may induce priming effects and individuals who are frequently exposed to such ads may develop stronger mental associations between the advertised product and the health context in which it is presented (Higgins, 1996; Harris *et al.*, 2009). Thus, when individuals are exposed to different food options, they may underestimate the unhealthy impact of products associated with healthwashed claims and may therefore be more likely to choose these products. This is especially true for food choices in grocery stores, in which choices are often made spontaneously (typically in under a second) or under time pressure (Verbeke, 2008; Cohen and Babey, 2012). Under such circumstances, consumers often rely on heuristics, such as the familiarity of the product and the associations, which come to their minds (e.g. healthiness).

### The effect of prior health information

Existing research indicates that individuals tend to allocate a minimum of resources to cognitively process food-related information, such as ad content (Rosbergen *et al.*, 1997; Verbeke, 2008). They may hence often rely on a peripheral route of processing (Petty *et al.*, 1983; Sheeran *et al.*, 2013). This may be especially true for less informed individuals, who have less prior knowledge and are less attentive to persuasive health messages. Thus, they may be less equipped and motivated to critically question encountered health claims in ads compared with highly informed individuals (Petty *et al.*, 1983; Nutbeam, 2000). In this context, the provision of health information becomes important so that consumers can quickly judge the accuracy of a health claim.

However, existing research indicates that people’s nutrition knowledge and assessment skills are generally low (e.g. Parmenter *et al.*, 2000; Grunert *et al*., 2011). For example, Parmenter *et al.* found that people mistakenly perceive muesli bars, which are usually high in added sugar, as healthy snacks (Parmenter *et al.*, 2000). The authors argue that this finding ‘might be attributable to marketing, presenting an image of muesli bars as a “healthy” alternative to more fattening snacks and that the pervasiveness of the error could indicate that advertising is used by many people as a source of nutrient information’ (Parmenter *et al.* 2000, p. 170). In other words, many people may not question misleading health messages in ads, but may instead use that information to make diet choices.

As a result, the provision of additional health-related nutritional information prior to ad exposure may influence how individuals judge healthwashing attempts. For example, such additional information may boost and activate knowledge about the consequences of sugar consumption and thus induce resistance to persuasive attempts. Inoculation theory assumes that through exposing individuals to weakened arguments, they can be ‘immunized’ against future persuasive attempts (McGuire, 1964). Several studies have investigated the effect of inoculation messages on attitude resistance and, in sum, suggest a positive effect (Banas and Rains, 2010). The reason is that messages which include counter-facts and arguments may add to and activate existing knowledge, which can then be used to judge new incoming information ( Higgins, 1996 ). For example, if consumers add new and activate existing knowledge about the unhealthy nature of sugar, they may engage in a more systematic processing, in which they elaborate on and disguise the persuasive intention behind misleading ads for high-sugar products (Petty *et al.*, 1983; Sheeran *et al.*, 2013).

The existing literature also suggests that once individuals have uncovered a misleading intend of an ad, that they may develop more negative attitudes towards consuming the product (Burton *et al.*, 2000; Schmuck *et al*., 2018). This is because individuals detect a discrepancy between the health claims in ads and the factual nutritional value of the products. As a result, individuals may become more reluctant to buy, personally use or distribute the advertised products. Following this reasoning, individuals high in healthwashing perceptions may report a more negative attitude towards product consumption.

Based on the backgrounds discussed above, the following two hypotheses are proposed:

*H1: Exposure to healthwashed ads only increases healthwashing perceptions if individuals receive additional health-related nutritional information prior to exposure.*

*H2: Healthwashing perceptions induce a more negative attitude towards consuming the advertised products.*

## METHODS

### Data collection

**Fig. 1:**
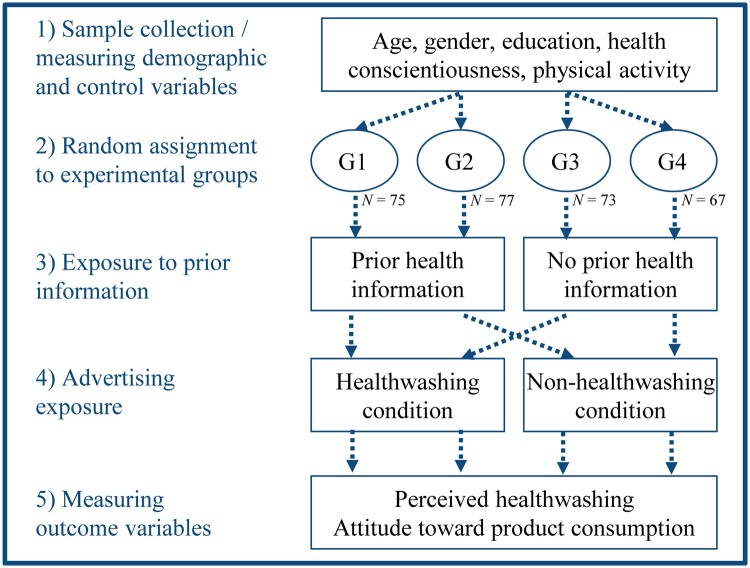
Flowchart of experimental design and implementation. In Stage 1, we collected the sample and measured demographic characteristics as well as the control variables. In Stage 2, we randomly assigned the participants of our sample to four groups. In Stage 3, we exposed the groups to either prior health information or not. In Stage 4, we exposed each group to ads either in the healthwashing or the non-healthwashing condition. Finally, in Stage 5, we measured the outcome variables across the whole sample.

We conducted an online experiment employing a 2 (exposure to healthwashed vs. non-healthwashed ads) × 2 (no prior health information vs. prior health information) experimental design. The recruitment of participants was implemented by the private company *Survey Sampling International*. We defined population-based quotes for age, sex and education. The final sample consisted of 292 individuals, 52.40% of which were males, 20.21% had a college-bound high school degree and 18.15% had a college degree. The mean age was 41.25 (SD = 13.59, min = 18 and max = 65). We only sampled individuals aged up to 65 years because the experiment was part of a larger online study that mainly focused on social media use (which is less frequent in older adults). Thus, it should be noted that we can only make conclusions for the specific age group used in our sample and that individuals aged >65 years may react differently to our stimulus material. Respondents completed the questionnaire via personal computers (54%), their smartphones (40%) or tablets (6%). To test whether our stimulus material could be deemed effective, we added treatment check tests, which we report below. The online survey was administered using the Unipark survey tool. Figure 1 provides an overview of the research design and data collection process.

### Stimulus material

The authors developed the stimulus material using existing professional advertising material and additional content to create externally and internally valid fake ads for the high-sugar products Ovomaltine spread, Nusspli spread, Lion Cereals and Mezzo Mix (see Supplementary Material for examples).

#### Prior health information

We exposed individuals either to an informational text about sugar levels and effects (prior health information condition) or to a text about different types of vegan lifestyles (no prior health information condition). In the prior health information condition, individuals were informed about the WHO’s (WHO, 2015) recommendation about individuals’ daily sugar intake, their actual sugar intake, some information about the sugar level in different products and how the overconsumption of sugar is related to obesity and diabetes. The text appeared on an official government Website (gesundheit.gv.at, see Supplementary Material). In the no prior health information condition, we exposed individuals to a text about current vegan trends and different types of vegetarian nutrition, but did not provide any health-related nutritional information. The text was of the same length and appeared in the same layout as the text in the prior health information condition (see Supplementary Material). The primary intent to provide a text in the no prior health information condition was to keep the cognitive effort constant across groups.

#### Healthwashing

We exposed individuals to four ads of high-sugar products. The ads either contained inappropriate health claims (healthwashing condition) or did not contain any of such claims (non-healthwashing condition). The healthwashed ads contained both visual (sports) and textual (e.g. ‘Stay active’) health claims, whereas the non-healthwashing condition did not make any health-related associations. After exposure to the ads, participants were asked questions about whether the ads contained inappropriate health claims and how frequently the advertised products should be consumed.

### Measures

If not stated otherwise, all of the following items were measured on 7-point Likert-type scales. *Perceived healthwashing* (*α*  = 0.89, *M *=* *5.10, SD = 1.54) was measured with five items asking participants whether they agreed that the presented ads (i) misled with words about their health features; (ii) misled with visuals or graphics about their health features; (iii) exaggerated the products’ actual health value; (iv) masked important information, making the health impact sound better than it is; or (v) did not tell the truth about the products’ impact on health (1 = don’t agree and 7 = agree, based on the scale of perceived greenwashing by Chen and Chang, 2013). *Attitude towards product consumption* (*α*  =  0.90, *M *=* *2.46, SD = 1.21) was measured with four questions, one for each product, asking participants how often people should eat or drink the advertised products, (i) Ovomaltine, (ii) Lion, (iii) Mezzo Mix or (iv) Nusspli. The participants responded on a 7-point scale, which included the following options: never, once a month, two or three times per month, once a week, two or three times per week, daily or multiple times per day (1 = never and 7 = multiple times per day). We assess this general measure rather than brand evaluation or purchase intention, for it may not only capture whether an individual may buy the products for personal intake but also share the products with other individuals in their environment (e.g. children).

*Health conscientiousness* *(α* = 0.92; *M* = 4.91, SD =1.41) was measured by asking participants whether they agreed that they (i) were alert to changes in their health, (ii) reflected about their health a lot, (iii) were always aware of their physical well-being or (iv) were generally aware of the state of their health (1 = don’t agree and 7 = agree, see Willis and Stafford, 2016). *Physical activity* *(M* = 39.18, SD = 27.04) was measured by asking participants how many days a week they performed (i) strenuous exercise (e.g. jogging), (ii) moderate exercise (e.g. badminton) or (iii) mild exercise (e.g. golf). The activities were weighted (9 = strenuous, 5 = moderate and 3 = mild) and then added up to a single scale (see Shephard, 1997). The control variables were assessed prior to exposure to the stimulus material.

### Statistical analysis

We used the statistics programme R to run our analyses. If not stated otherwise, we used ordinary least squares (OLSs) regressions for the randomization check, the treatment check and hypotheses testing. We used this approach because we predicted variables which are commonly treated as continuous variables, such as 5-point Likert scales. However, attitudes toward product consumption were measured with labelled, ordinal scales. Ordinal dependent scales can be used in OLS regression, too, if the distances between the single points of the scales can be treated as equal from a theoretical perspective (Allison, 1999). We are confident that the choice of our labels justifies treating the variable as continuous. Furthermore, treating the variable as continuous also allows us to combine the four questions for each food product to a single mean scale. However, to avoid analytical errors, we also ran an additional analysis to test whether our results remain robust using ordinal logistic regression.

In order to perform mediation analysis and calculate indirect paths, we used the mediation package in R and calculated quasi-Bayesian confidence intervals based on 5.000 Monte Carlo simulations (Tingley *et al.*, 2014).

## RESULTS

### Randomization and treatment checks

We performed randomization checks to test for differences in the distribution of health conscientiousness, physical activity, age, gender and education across the four experimental groups (for gender and education, binary logistic regressions were used). We did not find any significant differences. Hence, randomization was considered to be successful. Furthermore, the experimental conditions consisted of a similar number of individuals (see Figure 1). We also implemented treatment checks which indicate that those in the healthwashing condition recognized the health cues in the ads and that participants in the prior health information condition had higher sugar-related nutrition knowledge than those in the no prior health information condition. Furthermore, we tested whether the presented ads were perceived as equally professional across all conditions (see Supplementary Table S1).

### Hypotheses testing

**Fig. 2:**
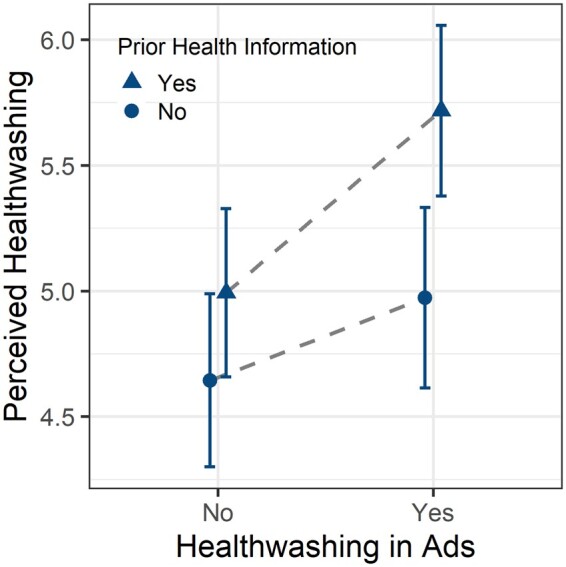
Mean values of perceived healthwashing across experimental groups (error bars indicate 95% CI). The graph is based on Model 1 in Table 1.

Model 1 in Table 1 shows the OLS regression results of the treatment effects without any control variables added. The coefficients indicate the difference between the mean values of the experimental groups, with the prior health information/healthwashing condition serving as the reference group (Figure 2). The results indicate that when no prior health information had been provided, the mean value differences in healthwashing perceptions between the healthwashing condition and the non-healthwashing condition was weak and insignificant (*b *=* *0.33, *p *=* *0.19). More importantly, we found that within the prior health information condition, individuals who were exposed to healthwashing scored significantly higher on perceived healthwashing than individuals who were exposed to no healthwashing. The average difference between the two groups is 0.73 points on the 7-point scale (*p *=* *0.003). Hence, our data provide support for H1. Furthermore, the results also indicate that individuals in the prior health information/healthwashing condition scored significantly higher on perceived healthwashing than individuals in the no prior health information/healthwashing condition (differences in means = 0.74, *p *=* *0.003) and individuals in the no prior health information/non-healthwashing condition (differences in means = 1.07, *p *<* *0.001).

**Table 1:** OLS regressions predicting perceived healthwashing (Models 1 and 2) and attitude toward product consumption (Model 3)

	M1: perceived healthwashing	M2: perceived healthwashing	M3: perceived APC^a^
	*b* (SE)	*b* (SE)	*b* (SE)
Age		0.004 (0.01)	0.0004 (0.01)
Highly educated		0.47* (0.23)	−0.34 (0.18)
Medium educated		0.09 (0.23)	−0.07 (0.18)
Male		−0.24 (0.17)	0.27* (0.14)
Health consciousness		0.15* (0.06)	−0.12* (0.05)
Physical activity		0.01 (0.003)	0.001 (0.003)
nHW/nPHI (vs. HW/PHI)^b^	−1.07*** (0.25)	−1.05*** (0.24)	0.08 (0.20)
nHW/PHI (vs. HW/PHI)^b^	−0.73** (0.24)	−0.73** (0.24)	−0.32 (0.19)
HW/nPHI (vs. HW/PHI)^b^	−0.74** (0.25)	−0.75** (0.25)	0.01 (0.20)
Perceived healthwashing			−0.16*** (0.05)
Constant	5.72*** (0.17)	4.86*** (0.50)	3.54*** (0.46)
Observations	292	292	292
Adjusted *R*^2^	0.06	0.10	0.09

a APC, attitude toward product consumption.

b HW, healthwashing condition; nHW, non-healthwashing condition; nPHI, no prior health information condition; PHI, prior health information condition. The models show unstandardized coefficients along with standard errors in parentheses.

Significant level: *p < 0.05; **p < 0.01; and ***p < 0.001.

The mean values along with their 95% CIs are plotted in Figure 2 (based on Table 1, model 1). Again, the graph shows that individuals in the prior health information/healthwashing condition scored substantially and significantly higher on perceived healthwashing than individuals in other groups. Its CIs do not overlap with those of any other group. This provides strong evidence that higher levels of health information enable individuals to detect inappropriate health claims. This effect remains also robust and significant in a larger model, controlling for demographics, health consciousness and physical activity (Model 2).

H2 assumed that perceived healthwashing would be negatively related with individuals’ attitudes towards product consumption. To investigate this question, we have included potentially influencing control variables in the model, including demographics, health consciousness and physical activity (see Model 3 in Table 1). The results lend support for H2. A one-unit increase in perceived healthwashing was related to a 0.16 unit decrease in individuals’ attitudes towards product consumption (*p *<* *0.001; both variables were measured on a 7-point scale). Because attitudes towards product consumption are composed of four labelled, ordinal items, we also ran the analysis using four separate ordinal logistic regression models to predict each item (see Supplementary Table S2). We find significant relations between perceived healthwashing and all four items. Thus, we are confident that our results are robust.

To test the indirect paths outlined in our theoretical model, we calculated the indirect effects (mediation analysis) based on Models 2 and 3. Model 2 includes the same set of control variables as Model 3; therefore, we use Model 2 rather than Model 1. The results indicate that healthwashing perceptions mediate the experimental effects on attitude towards consuming the advertised products. More precisely, compared with all other three experimental conditions, the prior health information/healthwashing condition had a negative effect on attitude towards product consumption via increased healthwashing perceptions. We found significant indirect effects of the prior health information/healthwashing condition compared with the no prior health information/non-healthwashing condition (*b* = −0.17, *p *<* *0.001, lower CI = −0.06, upper CI = −0.32), the prior health information/non-healthwashing condition (*b* = −0.12, *p *<* *0.01, lower CI = −0.03, upper CI = −0.25) and the no prior health information/healthwashing condition (*b* = −0.12, *p *<* *0.01, lower CI = −0.03, upper CI = −0.25).

## DISCUSSION

This study has shown that informing individuals about sugar levels in food products and their negative health effects prior to choice can make individuals more aware of inappropriate health claims used by food companies to disguise the unhealthy nature of their products. In fact, when we did not provide health information prior to ad exposure, individuals were unable to distinguish between the healthwashed and the non-healthwashed ads. Only when we did provided specific health information prior to ad exposure, the healthwashed ads significantly boosted perceived healthwashing, which, in turn, was negatively related to attitudes towards consuming the advertised products.

These results have important theoretical and practical implications. First of all, our study contributes to the existing literature on the importance of individuals’ prior information levels when confronted with misleading health claims (Andrews *et al.*, 2000; Burton *et al.*, 2000). For example, Andrews *et al.* have shown that misleading health claims can create more favourable (but erroneous) attitudes toward unhealthy products (Andrews *et al.*, 2000). They also found evidence for a protective effect of knowledge and point to the importance of nutrition education efforts. Our results resonate with these findings and also highlight the link between health knowledge and other dimensions of a broader health literacy concept, such as individuals’ capacity to understand and appraise health claims in ads (Nutbeam, 2000; Truman *et al.*, 2020). Only if people can compare new information to mentally stored and activated health knowledge, they can understand and critically evaluate the new information and may act upon it.

In line with this, our study provides support for the notion that food ads may often influence consumers in a subtle way (Harris and Graff, 2012; Sheeran *et al.*, 2013). In fact, inappropriate links between positive health claims and unhealthy products in ads may often remain undetected. They may therefore have an implicit persuasive influence on consumption behaviour (Nairn and Fine, 2008) and lead to uninformed food choices (Jacquier *et al.*, 2012). Consumers may be especially receptive to such subtle influence if they have a low motivation to question healthwashing claims. For example, some people may use inappropriate health claims as a rational to establish a balance between the maximum pleasure and minimal harm of eating high-sugar products . Adopting this rational allows consumers’ to avoid cognitive dissonance linked to tempting, but potentially harmful behaviours, like eating high-sugar products (Hope *et al.*, 2018). However, if consumers are exposed to convincing facts before ad exposure, they may become more likely to engage in critical thinking and refuse to accept tempting healthwashing claims. Taken together, more studies are needed to fully understand the processing of healthwashing claims in ads.

From a practical perspective, the study has shown that individuals’ baseline information level may be low and that consumers may be ill equipped to appraise healthwashed claims in advertising. In this context, consumers might be highly susceptible to the healthwashing strategies and may make unsophisticated diet decisions based on biased perceptions about the nutritional quality of foods. However, this does not mean that the responsibility of achieving such skills should be delegated to the individual (Malloy-Weir *et al.*, 2016; Truman *et al.*, 2020). In fact, health care professionals and policy makers are well advised to take an active role in informing consumers about sugar levels and effects. This is critical because consumers are nowadays confronted with a myriad of conflicting information, often shared by unreliable sources and strategic ‘Astroturf’ organizations (Malhotra *et al.*, 2018). Thus, encountered information about nutrition becomes more and more difficult to judge and people have a high need to receive professional information from trusted sources (e.g. government or health care professional sources).

Against this background, policy makers need to create structural conditions, which allow citizens to acquire sufficient health knowledge and skills. First, policy makers can increase the accessibility of health information through educational measures, such as by providing more space for health and nutrition in the school curriculum. For example, Finland has introduced health education as an independent subject in basic education in 2004, and later extended the introduction to upper secondary education (Välimaa *et al.*, 2008). Increasing health literacy is a core goal of the Finish system and teachers in health education have to have a university background in this specific field (Paakkari and Paakkari, 2019). First evidence indicates that health education as a stand-alone school subjects may have positive influence on young people’s health attitudes and literacy (Aira *et al.*, 2014). In Austrian, health education is still considered as a cross-subject issue, which should be discussed across different subjects, such as biology or physical education.

Second, policy makers may run information campaigns to educate individuals about sugar levels and effects (Snyder, 2007). Such campaigns may include pointers on how to identify sugar levels in food labels and information on the differences between naturally occurring sugars in fibre-rich foods (e.g. fruits) and added (or concentrated, e.g. fruit juices) sugar in processed low-fibre foods. Third, our results also support previous calls for nutrition disclosures on the actual ads. For example, Burton *et al.* found that nutrition disclosures lead to negative product attitudes when the disclosure contradicts a health claim put forth in the ad (Burton *et al.*, 2000). In other words, nutrition disclosures can also increase consumers’ in situ information and support healthwashing detection. Finally, it should be mentioned that these awareness-building measures may also indirectly create public support for more strict policy regulations, such as sugar taxes (Scheelbeek *et al.*, 2019) or even sales and advertising restrictions (Malhotra *et al.*, 2018).

### Limitations

This study has some limitations. First, we provided the prior health information directly before ad exposure. Thus, individuals may have been specifically involved with the topic. Future research may also investigate how long such effects may endure and how we can make them robust over time (e.g. by using prolonged exposure designs). Second, we have introduced a new concept called healthwashing perceptions in this study and had to develop a new measure for this concept. We derived this measure from the literature, but its construct validity needs to be further tested. Third, we only tested the effects of print ads and the effects may differ for audiovisual or interactive advertising, such as in social media. Finally, future research may specifically focus on more vulnerable groups of individuals, such as less educated and younger people and how intervention materials may increase awareness about the persuasive nature of advertising content in these groups.

## CONCLUSIONS

These limitations notwithstanding, this study is one of the first studies that looks at consumers’ ability to critically appraise healthwashed claims in advertising. It unveiled that participants tend to be unable to distinguish healthwashed from non-healthwashed ads. Only additional information about nutritional properties before the ad exposure increased the critical appraisal of healthwashing. As the number and variety of health claims have been increasing, consumers should be able to evaluate and judge the encountered content. Only then will they be able to make informed decisions about their daily food consumption. Thus, increasing public access to health information may be an important measure to help consumers shield themselves from the myriad of inappropriate health claims in advertising and make more informed diet choices.

## Supplementary Data

Supplementary material is available at *Health Promotion International* online.

## Supplementary Material

daaa086_Supplementary_Data
