# Supplementary material for: Healthwashing in high-sugar food advertising: the effect of prior information on healthwashing perceptions in Austria
Source: Health Promot Int. 2020 Dec 8;36(4):1029–38. doi: 10.1093/heapro/daaa086 (PMC8405248; doi:10.1093/heapro/daaa086)
Supplement: daaa086_Supplementary_Data [file daaa086_supplementary_data.pdf]

Supplementary Material

Table S1: Measures and results of treatment checks (based on ordinary least squares regression analyses)

Measurement	Treatment check
Health cue recognition. To check whether our treatment was successful, we asked individuals whether the ads (1) *depicted sports or athletes* (M = 4.13, SD = 2.34) and (2) contained slogans that were related to *workout/energy/fitness* (M = 4.85, SD = 1.99). (1 = don't agree, 7 = agree).	We tested whether individuals in the healthwashing conditions recognised the health cues in the ads. Individuals in the healthwashing condition scored significantly higher on both questions than those in the non-healthwashing condition (Q1: b = 3.19, p < 0.001; Q2: b = 1.71, p < 0.001).
Sugar-related nutrition knowledge. We conducted a multiple choice test at the end of the experiment to assess their *sugar-related nutrition knowledge* (M = 3.51, SD = 1.08, range = 0–5), asking participants across all groups about the health facts we included in the text (i.e. 'What are potential health consequences of excessive sugar consumption', 'Which products contribute most to excessive sugar consumption?', 'What is the maximal amount of sugar intake for an adult person per day as recommended by the WHO?', 'How much is the actual consumption of sugar' and 'How much sugar is in a 500 ml bottle of coke?'). We created a cumulative index measuring sugar-related nutritional information level by summing up the correct responses.	We tested the effectiveness of the informational text we provided to individuals in the prior health information condition. Individuals in the prior health information condition (i.e. those who were exposed to the informational text) scored significantly higher in the sugar-related nutrition knowledge test than the individuals in the no prior health information condition (b = 1.18, p < 0.001).
Ad quality. Furthermore, we measured participants' *perceived quality of the ads* (α = 0.83; M = 4.13, SD = 1.50) by asking individuals whether they agreed that the ads were (1) well made, (2) convincing or (3) professionally designed (1 = don't agree, 7 = agree).	We checked whether the ads were perceived as equally professional across the healthwashing and non-healthwashing conditions. No significant differences occurred when comparing the perceived quality of the ads scores in the healthwashing condition against the score in the non-healthwashing condition (b = 0.24, p = 0.18).

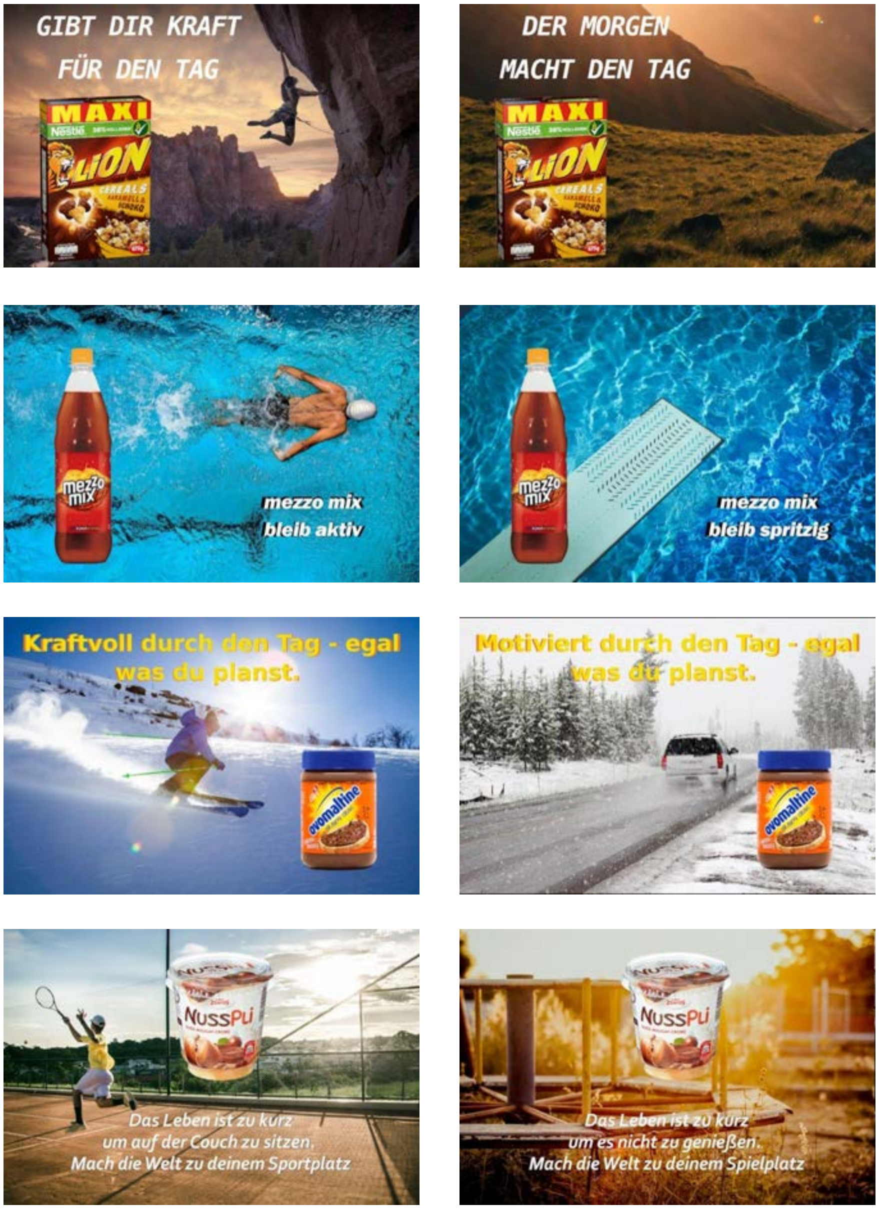

Text translation:
-Gives you energy for the day (left)
-The morning makes the day (right)

Text translation:
-Mezzo mix – stay active (left)
-Mezzo mix – stay bubbly (right)

Text Translation:
-Powerful through the day – no matter what you plan (left)
-Motivated throughout the day – no matter what you plan (right)

Text Translation:
-Life is too short to sit on your couch – make the world your own sports ground (left)
- Life is too short to sit on your couch – make the world your own playground (right)

Figure S1: Ads shown in the healthwashing condition (left) and the control condition (right)

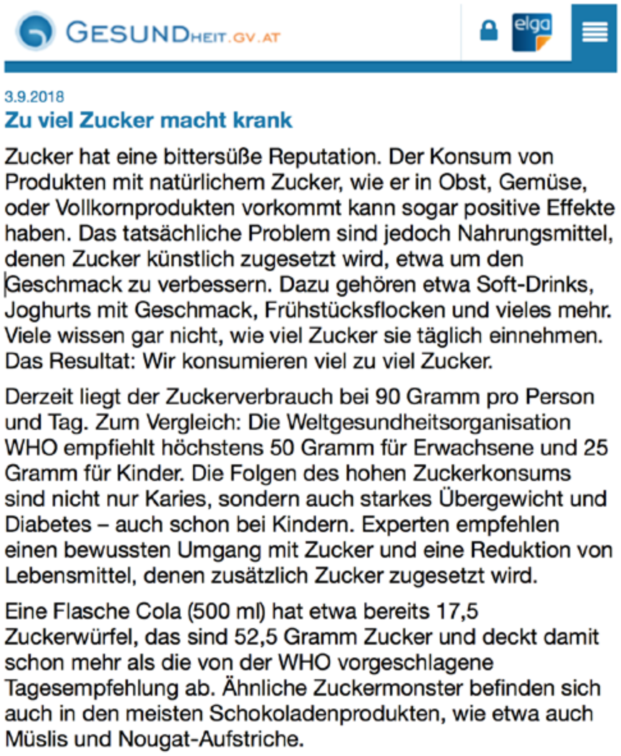

GESUNDheit.gv.at

3.9.2018

Zu viel Zucker macht krank

Zucker hat eine bittersüße Reputation. Der Konsum von Produkten mit natürlichem Zucker, wie er in Obst, Gemüse, oder Vollkornprodukten vorkommt kann sogar positive Effekte haben. Das tatsächliche Problem sind jedoch Nahrungsmittel, denen Zucker künstlich zugesetzt wird, etwa um den Geschmack zu verbessern. Dazu gehören etwa Soft-Drinks, Joghurts mit Geschmack, Frühstücksflocken und vieles mehr. Viele wissen gar nicht, wie viel Zucker sie täglich einnehmen. Das Resultat: Wir konsumieren viel zu viel Zucker.

Derzeit liegt der Zuckerverbrauch bei 90 Gramm pro Person und Tag. Zum Vergleich: Die Weltgesundheitsorganisation WHO empfiehlt höchstens 50 Gramm für Erwachsene und 25 Gramm für Kinder. Die Folgen des hohen Zuckerkonsums sind nicht nur Karies, sondern auch starkes Übergewicht und Diabetes – auch schon bei Kindern. Experten empfehlen einen bewussten Umgang mit Zucker und eine Reduktion von Lebensmittel, denen zusätzlich Zucker zugesetzt wird.

Eine Flasche Cola (500 ml) hat etwa bereits 17,5 Zuckerwürfel, das sind 52,5 Gramm Zucker und deckt damit schon mehr als die von der WHO vorgeschlagene Tagesempfehlung ab. Ähnliche Zuckermonster befinden sich auch in den meisten Schokoladenprodukten, wie etwa auch Müslis und Nougat-Aufstriche.

Figure S2: Sugar-related text (high information condition)

Translation:

Too much sugar makes you sick

Sugar has a bittersweet reputation. The consumption of products with natural sugar, as found in fruits, vegetables or whole grains, can even have positive effects. The real problem, however, are foods to which sugar is artificially added, for example to improve the taste. These include soft drinks, yoghurts with flavour, cereals and many more. A lot of people do not know how much sugar they use daily. The result: we consume too much sugar.

The sugar consumption per person per day is now up to 90 g. By comparison, the World Health Organization recommends a maximum of 50 g for adults and 25 g for children. The consequences of high-sugar intake are not only dental caries but also obesity and diabetes, even in children. Experts recommend a conscious handling of sugar and a reduction of foods that contain added sugar.

A bottle of cola (500 ml) contains about 17.5 sugar cubes, i.e. 52.5 g of sugar. This already exceeds the daily recommendation by the WHO. Similar sugar monsters are found in most chocolate products, such as cereals and nougat spreads.

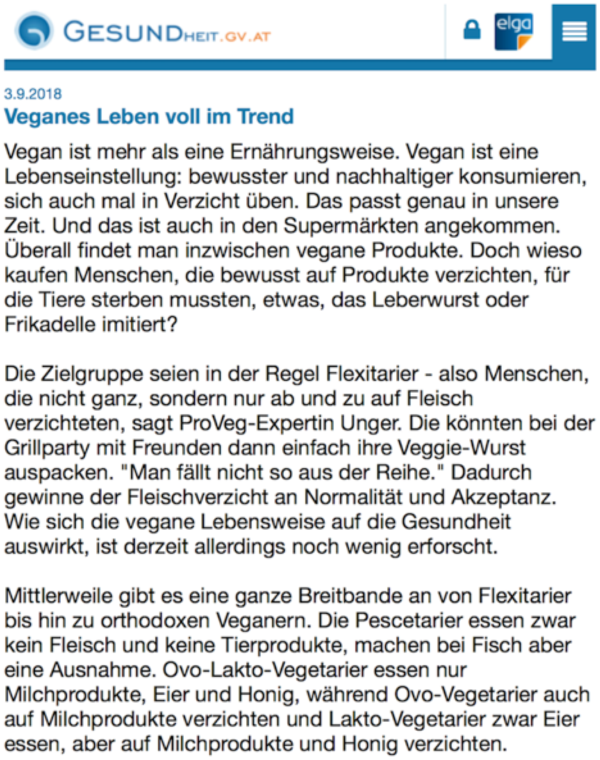

GESUNDHEIT.GV.AT

3.9.2018

Veganes Leben voll im Trend

Vegan ist mehr als eine Ernährungsweise. Vegan ist eine Lebenseinstellung: bewusster und nachhaltiger konsumieren, sich auch mal in Verzicht üben. Das passt genau in unsere Zeit. Und das ist auch in den Supermärkten angekommen. Überall findet man inzwischen vegane Produkte. Doch wieso kaufen Menschen, die bewusst auf Produkte verzichten, für die Tiere sterben mussten, etwas, das Leberwurst oder Frikadelle imitiert?

Die Zielgruppe seien in der Regel Flexitarier - also Menschen, die nicht ganz, sondern nur ab und zu auf Fleisch verzichteten, sagt ProVeg-Expertin Unger. Die könnten bei der Grillparty mit Freunden dann einfach ihre Veggie-Wurst auspacken. "Man fällt nicht so aus der Reihe." Dadurch gewinne der Fleischverzicht an Normalität und Akzeptanz. Wie sich die vegane Lebensweise auf die Gesundheit auswirkt, ist derzeit allerdings noch wenig erforscht.

Mittlerweile gibt es eine ganze Breitbande an von Flexitarier bis hin zu orthodoxen Veganern. Die Pescetarier essen zwar kein Fleisch und keine Tierprodukte, machen bei Fisch aber eine Ausnahme. Ovo-Lakto-Vegetarier essen nur Milchprodukte, Eier und Honig, während Ovo-Vegetarier auch auf Milchprodukte verzichten und Lakto-Vegetarier zwar Eier essen, aber auf Milchprodukte und Honig verzichten.

Figure S3: Sugar-unrelated text (baseline information condition)

Translation:

Vegan life is very trendy

Vegan is more than a diet. Vegan is a way of life: to consume more consciously and more sustainably, sometimes to renounce oneself. This fits exactly in our time. Moreover, the vegan lifestyle has also arrived in the grocery stores. Everywhere you can find vegan products. But why do people who avoid products for which animals had to die for seek products that mimic sausages or meatballs?

The target group are usually flexitarians—people who do not completely, but only occasionally, abstain from eating meat, says ProVeg expert Unger. They could simply unpack their veggie sausage at the barbecue party with friends. ‘You do not fall out of line’. As a result, the occasional renunciation of meat gains normality and acceptance. How the vegan lifestyle may affect health, however, is currently under research.

Today, there is a whole range of vegetarians, from flexitarians to orthodox vegans. For example, pescetarians eat only fish, but do not eat meat from any other animals. Ovo-lacto vegetarians eat only dairy products, eggs and honey, while ovo-vegetarians do omit dairy products and lacto-vegetarians eat eggs, but do avoid dairy products and honey.

Table S2: Ordinal logistic regressions predicting perceived attitude toward product consumption for each product in the stimulus material

	Lion	Mezzo Mix	Ovomaltine	Nusspli
Age	0.01 (0.01)	-0.001 (0.01)	-0.002 (0.01)	-0.01 (0.01)
Highly Educated	-0.63* (0.29)	-0.43 (0.32)	-0.78* (0.30)	-0.36 (0.30)
Medium Educated	-0.42 (0.28)	-0.08 (0.30)	-0.09 (0.28)	0.19 (0.28)
Male	0.41^{+} (0.21)	0.43^{+} (0.23)	0.20 (0.21)	0.41^{+} (0.22)
Health Consciousness	-0.16^{+} (0.08)	-0.24** (0.09)	-0.15^{+} (0.08)	-0.16^{+} (0.08)
Physical Activity	-0.0001 (0.004)	0.004 (0.005)	-0.001 (0.004)	0.003 (0.004)
nHW/nPHI (vs. HW/PHI)[b]	0.03 (0.30)	0.05 (0.32)	0.001 (0.30)	0.07 (0.31)
nHW/PHI (vs. HW/PHI)[b]	-0.50^{+} (0.30)	-0.43 (0.33)	-0.33 (0.31)	-0.60 (0.31)
HW/nPHI(vs. HW/PHI)[b]	0.35 (0.31)	0.12 (0.33)	-0.01 (0.31)	0.11 (0.31)
Perceived Healthwashing	-0.27*** (0.08)	-0.19* (0.08)	-0.18* (0.08)	-0.23** (0.08)
Constants				
1|2[c]	-2.53 (0.72)	-1.48 (0.78)	-2.65 (0.72)	-2.17 (0.76)
2|3[c]	-1.77 (0.72)	-0.79 (0.77)	-1.74 (0.71)	-1.16 (0.75)
3|4[c]	-0.94 (0.72)	0.06 (0.77)	-0.81. (0.70)	-0.11 (0.75)
4|5[c]	0.46 (0.72)	1.25 (0.79)	0.76. (0.72)	1.57 (0.78)
5|6[c]	1.87 (0.75)	2.67 (0.89)	2.16 (0.78)	2.64 (0.86)
Observations	292	292	292	292
Log Likelihood	-462.19	-389.63	-456.97	-420.67

Note: [a]APC = Attitude toward product consumption. [b] HW = healthwashing condition, nHW = non-healthwashing condition, nPHI = no prior health information condition, PHI= prior health information condition. [c]Refers to the level of the dependent variable: 1 = never, 2 = once a month, 3 = two or three times per month, 4 = once a week, 5 = two or three times per week, 6 = daily or multiple times per day.
The models show unstandardized coefficients along with standard errors in parentheses.
Sig. Level: + p<0.1; * p<0.05; ** p<0.01; *** p<0.001
